# Author Correction: Biologically driven DOC release from peatlands during recovery from acidification

**DOI:** 10.1038/s41467-018-07157-2

**Published:** 2018-10-31

**Authors:** Hojeong Kang, Min Jung Kwon, Sunghyun Kim, Seunghoon Lee, Timothy G. Jones, Anna C. Johncock, Akira Haraguchi, Chris Freeman

**Affiliations:** 1 School of Civil and Environmental Engineering, Yonsei University, Seoul, 03722 Korea; 2 School of Natural Sciences, Bangor University, Bangor, LL57 2UW UK; 3 The University of Kitakyushu, Kitakyushu, 802-8577 Japan; 4 Present Address: Korea Polar Research Institute, Inchon, 21990 Korea; 5 Present Address: Smithsonian Environmental Research Center, Edgewater 21037-0028, MD USA; 6 Present Address: Shine Biopharm Inc., Seoul, 06536 Korea

Correction to: *Nature Communications*; 10.1038/s41467-018-06259-1; published online 18 September 2018

The original version of this Article contained an error in the Acknowledgements, which incorrectly omitted from the end the following: ‘In particular, we thank the staff of the Centre for Ecology and Hydrology (including A. Burden, N. Ostle and C. Evans) in relation to a NERC grant involving CF & TJ (NE/E011748/1; 2007–2010), which established the sites from which the UK samples were subsequently collected.’ This has been corrected in both the PDF and HTML versions of the Article.

