# Supplementary material for: Biologically driven DOC release from peatlands during recovery from acidification
Source: Nat Commun. 2018 Sep 18;9:3807. doi: 10.1038/s41467-018-06259-1 (PMC6143518; doi:10.1038/s41467-018-06259-1)
Supplement: Supplementary file 1 — Supplementary Information [file 41467_2018_6259_MOESM1_ESM.pdf]

Supplementary Information

Title: Biologically driven DOC release from peatlands during recovery from acidification

Authors: Kang *et al.*

Supplementary Tables

Supplementary Table 1. A summary of environmental and chemical properties of the 7 peatland sites.

	Yong	Odae	Mujechi	Tadewara	Kalimantan	Migneint	Peaknaze
Location	38°21′ N<br>128°13′ E	37°80′ N<br>128°54′ E	35°26′ N<br>129°8′ E	32°39′ N<br>131°29′ E	1°88′ S<br>113°53′ E	52°96′ N<br>3°82′ W	53°47′ N<br>1°92′ W
Mean annual temperature (°C)	14.4	7.4	10.5	8.0	27.5	8.6	8.0
Annual precipitation (mm)	1,176	2,100	1,155	2,700	2,765	2,200	1,000
OM (%)	51.0 ± 25.9	62.5 ± 14.6	41.0 ± 19.5	91.4 ± 1.6	88.2 ± 2.8	96.8 ± 1.4	92.0 ± 2.6
pH	5.41 ± 0.23	5.94 ± 0.25	4.97 ± 0.47	5.17 ± 0.14	4.45 ± 0.09	4.32 ± 0.13	3.95 ± 0.15
DOC (mg L^{-1})	8.30 ± 4.06	5.98 ± 4.51	6.78 ± 4.18	2.30 ± 0.21	39.79 ± 1.00	26.36 ± 9.59	53.20 ± 3.76
Phenolics (mg L^{-1})	1.84 ± 1.54	2.14 ± 0.97	0.82 ± 1.26	2.10 ± 0.12	5.19 ± 1.74	5.31 ± 2.32	8.04 ± 3.99

Supplementary Table 2. Accession codes for the dataset of 16S rDNA gene amplicon sequences reported in this study.

Samples	GenBank Accession Numbers	BioSample ID
Mujechi wetland soil_center	KCEQ00000000	SAMN09662675
Mujechi wetland soil_middle	KCEO00000000	SAMN09662676
Mujechi wetland soil_edge	KCEP00000000	SAMN09662636
Mujechi upland soil	KCEN00000000	SAMN09662677
Yong wetland soil_center	KCEL00000000	SAMN09662690
Yong wetland soil_middle	KCEI00000000	SAMN09662692
Yong wetland soil_edge	KCEJ00000000	SAMN09662693
Yong upland soil	KCEK00000000	SAMN09662695
Odae wetland soil_center_AUG	KCEM00000000	SAMN09662696
Odae wetland soil_middle_AUG	KCDZ00000000	SAMN09662697
Odae wetland soil_edge_AUG	KCEH00000000	SAMN09662698
Odae upland soil_AUG	KCEB00000000	SAMN09662700
Odae wetland soil_center_OCT	KCDW00000000	SAMN09662702
Odae wetland soil_middle_OCT	KCEE00000000	SAMN09662701
Odae wetland soil_edge_OCT	KCDY00000000	SAMN09662703
Odae upland soil_OCT	KCEA00000000	SAMN09662704
Migneint peat_control1	KCEC00000000	SAMN09662713
Migneint peat_control2	KCEG00000000	SAMN09662714
Migneint peat_control3	KCDX00000000	SAMN09662715
Migneint peat_control4	KCEF00000000	SAMN09662716
Migneint peat_acid1	KCED00000000	SAMN09662718
Migneint peat_acid2	KCDV00000000	SAMN09662725
Migneint peat_acid4	KCDU00000000	SAMN09662730
Migneint peat_acid3	KCDT00000000	SAMN09662729
Migneint peat_alkaline1	KCDH00000000	SAMN09662731
Migneint peat_alkaline2	KCDO00000000	SAMN09662732
Migneint peat_alkaline3	KCDG00000000	SAMN09662767
Migneint peat_alkaline4	KCDI00000000	SAMN09662768
Peaknaze peat_control1	KCDJ00000000	SAMN09662769
Peaknaze peat_control2	KCDL00000000	SAMN09662770
Peaknaze peat_control3	KCDM00000000	SAMN09662772
Peaknaze peat_acid1	KCDF00000000	SAMN09662788
Peaknaze peat_acid2	KCDS00000000	SAMN09662789
Peaknaze peat_acid3	KCDR00000000	SAMN09662790
Peaknaze peat_acid4	KCDP00000000	SAMN09662809
Peaknaze peat_alkaline1	KCDQ00000000	SAMN09662810
Peaknaze peat_alkaline2	KCDN00000000	SAMN09662811
Peaknaze peat_alkaline3	KCDK00000000	SAMN09662812
Peaknaze peat_alkaline4	KCDE00000000	SAMN09662815

Supplementary Figures

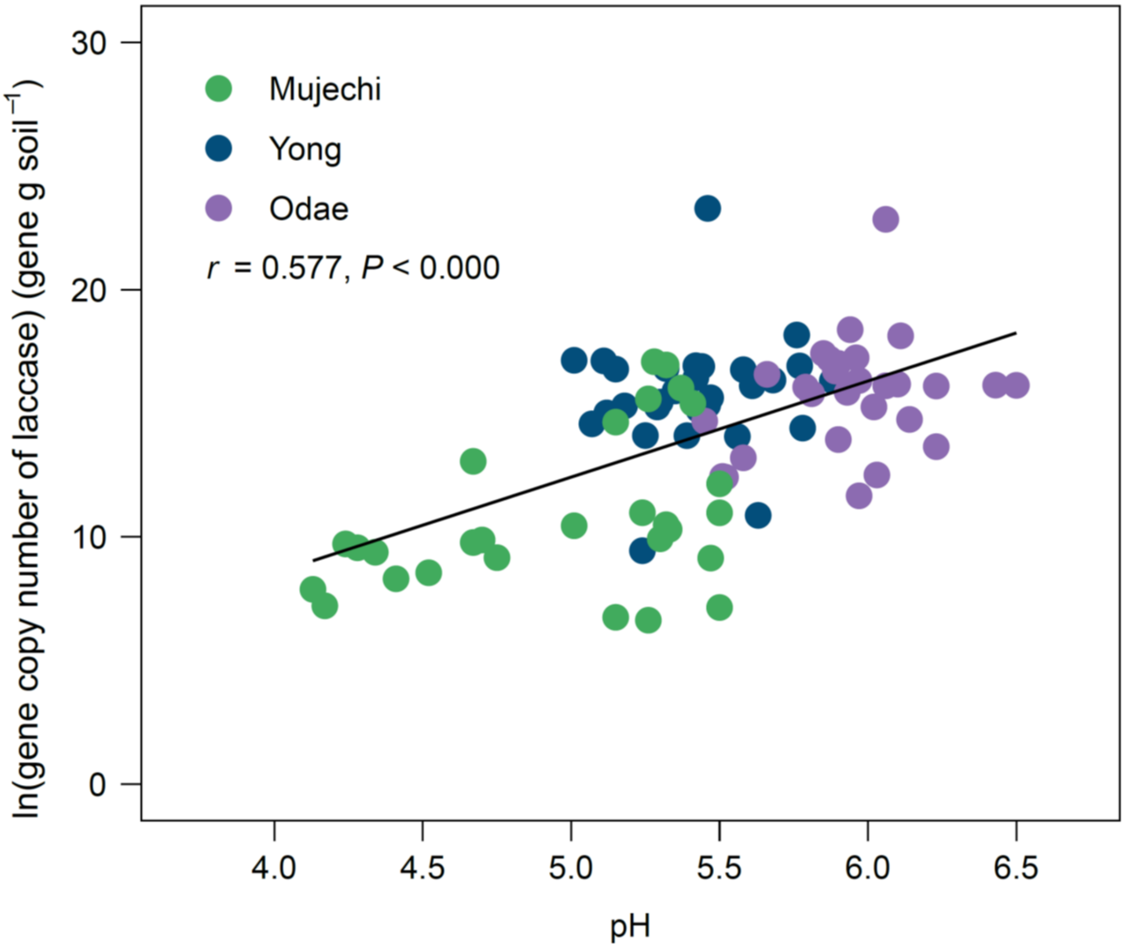

Supplementary Figure 1. A positive correlation between pH and the logarithm of gene copy number of laccase in three peatlands.

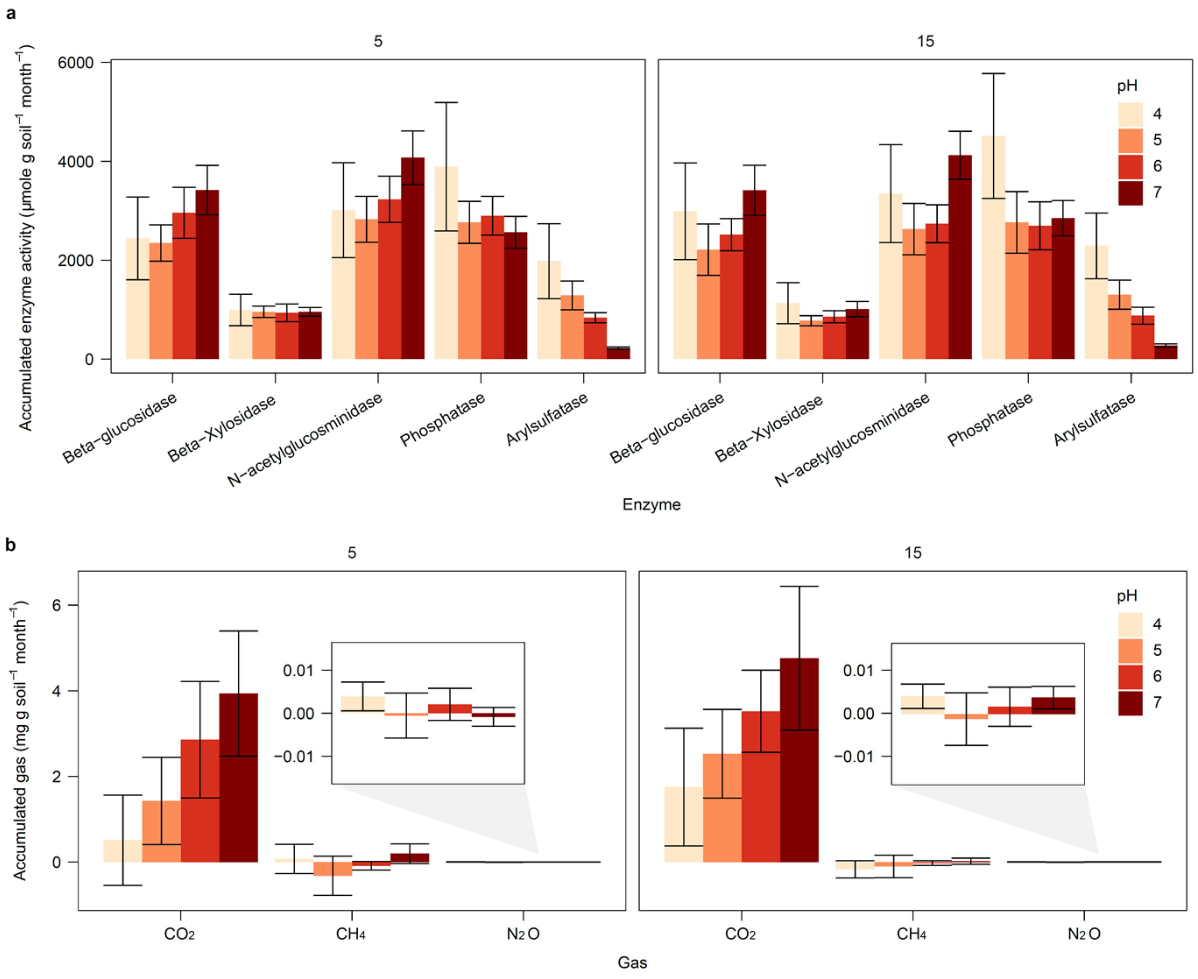

Supplementary Figure 2. Accumulative hydrolase activities, phenolic concentration and trace gas emissions from peat soils incubated at different pH conditions (4, 5, 6 and 7) and temperatures (5 ºC and 15 ºC) for a month. Error bars indicate standard of error of the means.
